# Effects and Safety of Convalescent Plasma Administration in a Group of Polish Pediatric Patients with COVID-19: A Case Series

**DOI:** 10.3390/life11030247

**Published:** 2021-03-17

**Authors:** Paweł Małecki, Kamil Faltin, Anna Mania, Katarzyna Mazur-Melewska, Agnieszka Cwalińska, Anna Zawadzka, Alicja Bukowska, Katarzyna Lisowska, Katarzyna Graniczna, Magdalena Figlerowicz

**Affiliations:** 1 Department of Infectious Diseases and Child Neurology, Poznan University of Medical Sciences, Szpitalna Street 27/33, 60-572 Poznań, Greater Poland Voivodeship, Poland; pmalecki@ump.edu.pl (P.M.); FaltinKamil@interia.pl (K.F.); amania@ump.edu.pl (A.M.); katarzynamelewska@ump.edu.pl (K.M.-M.); agnieszka.cwalinska@ump.edu.pl (A.C.); 2 Regional Blood Center, Marcelinska Street 44, 60-354 Poznań, Greater Poland Voivodeship, Poland; anna.zawadzka@rckik.poznan.pl (A.Z.); alicja.bukowska@rckik.poznan.pl (A.B.); katarzyna.lisowska@rckik.poznan.pl (K.L.); katarzyna.graniczna@rckik.poznan.pl (K.G.)

**Keywords:** convalescent plasma, COVID-19, children, SARS-CoV-2, transfusion

## Abstract

Despite the enormous advances in knowledge about the SARS-CoV-2 infection, the optimal treatment for COVID-19 is still not well defined. The use of convalescent plasma seems to be a promising method of treatment but requires further evaluation. Although it is usually mild, in children with underlying chronic diseases, the course of SARS-CoV-2 infection may be very severe. We described a series of 13 pediatric patients (mean age 10.4 years, median 12) treated with convalescent plasma as a method of COVID-19 therapy. Medical history, with particular emphasis on comorbidities, clinical course, laboratory parameters, supportive treatment and virus elimination time, were analyzed. The mean hospitalization time was 22.6 days (median 20). The most common abnormalities included increased levels of C-reactive protein, D-dimer, and lymphopenia. Median time from symptom onset to convalescent plasma transfusion was 10.6 days (median 7 days). Six patients (46.2%) had a viral clearance on RT-PCR method from a nasopharyngeal swab within 3 days of transfusion, while in the remaining patients the mean elimination time was 12.1 days (median 6 days). Clinical improvement was achieved in all patients; no adverse effects were found in any of the cases. Convalescent plasma may be a promising treatment for COVID-19 in children.

## 1. Introduction

The first cases of the Severe Acute Respiratory Syndrome Coronavirus 2 (SARS-CoV-2) infections were reported in Wuhan, China, in December 2019. Over the next few months, the disease spread around the world, becoming “the Public Health Emergency of International Concern”, according to the World Health Organization (WHO) in 2020 [1]. The first case in Poland was diagnosed on March 4, 2020. The disease caused by SARS-CoV-2, called the Coronavirus Disease 2019 (COVID-19), leads to, in the most severe cases, acute lung injury, coagulation disorders, multi-organ failure, and ultimately death [2,3]. According to WHO reports, by mid-January 2021, there were 88 million reported cases and over 1.9 million deaths due to COVID-19 [4]. Based on ECDC (European Centre for Disease Prevention and Control) reports, the number of COVID-19 cases found in children under 15 in Europe amounted to over 12 million in 2020. In Poland, by November 2020, approximately 27,000 cases were found in the pediatric population [5,6].

The search for effective methods for the treatment and prevention of COVID-19 is a priority in the medical world. Observations to date indicate a milder course of infection in children. However, severe cases have also been described [7,8,9]. Children with COVID-19 and underlying chronic diseases are a particular challenge. The authors emphasize a more severe course of the acute phase of COVID-19 in children with obesity (significantly related to the need for mechanical ventilation, *p* = 0.03) or chronic kidney disease [8,10]. In comparative descriptions of infections in children and adults, the clinical course in the pediatric population is described as milder, the outcome better, the mortality lower [11]. Nevertheless, later manifestations of the infection, such as MIS-C (Multisystem inflammatory syndrome in children) or the Kawasaki-like syndrome, are characteristic of the pediatric population [12]. Convalescent plasma (CP) has been used in the management of COVID-19 in adult patients, but data in children are limited [13]. In randomised trials involving the adult population, no significant efficacy of CP in the treatment of the SARS-CoV-2 infection was found, but clinical improvement and elimination of the virus have been reported in a single case and case series observations [14]. In attempts to elucidate the mechanisms of action of CP, specific antibodies against the receptor-binding domain (DBP), antibodies against full-length SARS-CoV-2 spikes, and antibodies against nucleocapsid (N) proteins were taken into account [15,16,17]. Studies suggest no direct relationship between the neutralising efficacy of CP and antibodies against SARS-CoV-2 [18].

In this paper, we describe a group of pediatric patients with a severe course of COVID-19, in whom we administered CP and assessed its effectiveness in the rapid elimination of the virus and improvement in the clinical condition.

## 2. Materials and Methods

Retrospective analysis of children hospitalized due to COVID-19 at the university’s infectious diseases department, from 15 February 2020 to 15 January 2021, was carried out. The described group included 13 patients (seven girls and 6sixboys)—all children treated with CP during the course of COVID-19.

We confirmed the SARS-CoV-2 infections by testing nasopharyngeal swabs using the real-time polymerase chain reaction (RT-PCR) method performed at the University Coronavirus Laboratory in each child. We used the COVID-19 Real-Time Multiplex RT-PCR Kit (Labsystem Diagnostics, Vantaa, Finland) and the 2019-Novel Coronavirus (2019-nCoV) Triplex RT-qPCR (Vazymebiotech, Nanjing, China) Kits. The examination was performed on the first day of hospitalisation and then, as standard, 3 days after the CP transfusion. In the event of a positive result on day 3, we repeated the test every 5–7 days until a negative result was obtained.

A detailed analysis of the medical history, especially of chronic diseases, has been carried out. We took the presence of symptoms such as fever > 38 degrees Celsius, cough, dyspnoea, headache, abdominal pain, muscle pain, anosmia, and dysgeusia into account. An attempt was made to determine a possible source of infection. We performed a detailed medical examination in all patients; vital parameters were monitored—temperature, heart rate, blood pressure, blood oxygen saturation level, and respiratory rate. The severity of the disease was defined as: mild (low inflammatory markers, normal lung ultrasound, good general condition), moderate (elevated inflammatory markers and/or abnormal lung ultrasound and/or moderate general condition, no indications for mechanical ventilation), and severe (need for mechanical ventilation). All patients had lung ultrasound examinations performed by a trained physician using a Samsung HS40 machine (convex probe CA2-8AD: 2–8 MHz, linear probe LA3-16AD: 3–16 MHz) and a chest X-ray examination.

Laboratory tests included: complete blood count with leukocyte differential, blood gases, the activity of alanine (ALT) and aspartate (AST) aminotransferase, lactate dehydrogenase (LDH), creatine kinase (CK), the concentration of C-reactive protein (CRP), ferritin, B-type natriuretic peptide (BNP), interleukin-6 (IL-6), fibrinogen, INR, and activated partial thromboplastin time (APTT). The laboratory parameters were evaluated using standard analysers. A blood culture was taken in all patients with a fever. In patients with lower respiratory tract infections, we performed PCR panel for other respiratory pathogens, including bocavirus, adenovirus, parainfluenza virus, rhinovirus, coronavirus, Epstein–Barr virus, *Mycoplasma pneumoniae, Staphylococcus aureus, Streptococcus pneumoniae*.

The regional blood donation centre employees were responsible for the qualification and collection of plasma from the volunteers. We transfused a volume of 5–10 mL/kg of the plasma of convalescent. Each patient received a single CP transfusion. We determined the serum IgG antibodies using the ELISA method (EUROIMMUN Medizinische Labordiagnostika AG, Lübeck, Deutschland). The reagent wells were coated with an S1 domain of the spike protein of SARS-CoV-2, expressed recombinantly in the human cell line HEK 293 (Human Embryonic Kidney cells).

Treatment with convalescent plasma was preceded by obtaining the legal guardian’s consent and the local Bioethics Committee at the Medical University (No. 376/20 of 15 May 2020, No. 732/20 of 4 November 2020 and No. 813/20 of 4 November 2020).

## 3. Results

All 13 patients in the analysed group suffered from chronic diseases. The mean age was 10.4 years (median 12, interquartile range (IQR) 6–16). The length of hospitalisation depended on the severity of the disease and complications, mean 22.6 days (range 8–59, median 20, IQR 15–31). We included a collective description of the group in Table 1.

In eight cases, the probable source of the infection was unknown; in four cases, it was a family member, and in one case, a friend. No patient had a history of international travel within 2 weeks before the onset of symptoms. Children were admitted to the hospital within 3 days, on average (median 2, IQR 2–5), after the first symptoms appeared (maximum 7 days).

The most common symptoms of COVID-19 were dyspnoea (four cases, 30.8%), cough (three cases, 23%), one patient reported abdominal pain, one a headache and muscle pain. We did not find any olfactory and taste disturbances in the analysed patients. Seven out of 13 children had a fever on admission, the highest temperature recorded was 39 degrees C.

On admission to the hospital, three patients had a blood oxygen saturation level of <92% (89% and 91%, respectively; patient No. 11 required immediate intubation due to respiratory failure).

Among the laboratory abnormalities typical for COVID-19, 7/13 (53.8%) patients had lymphopenia < 1.5 G/L, thrombocytopenia < 140 G/L was found in 3/13 (23%), increased LDH levels in 6/13 (46.2%), increased levels of ALT and AST in 4/13 (30.8%), increased levels of CRP in 8/13 (61.5%), troponin I in 5/13 (38.5%) and D-dimers in 9/13 (69.2%) patients. We presented a comparison of laboratory tests performed before and after the transfusion in Table 2.

The average time from onset of symptoms to CP transfusion was 10.6 days (median 7, IQR 5–12, range 3–37). Due to the severity of the infection, we treated 2 patients (numbers 4 and 12) with remdesivir (RDV). We administered glucocorticosteroids in nine patients: a patient with Acute Disseminated Encephalomyelitis (ADEM) was treated with methylprednisolone pulses of 30 mg/kg, a girl with nephrotic syndrome continued her previous prednisolone treatment, the remaining patients received a low-dose of dexamethasone (0.15 mg/kg). Four patients (No. 4, 5 and 11) required active oxygen therapy using a ventilator.

In six patients (46.2%), we obtained negative RT-PCR tests from nasopharyngeal swabs on day 3 after CP transfusion. In the remaining patients, the mean time to the RT-PCR test negativity was 12.1 days (range 6–30 days, median 6 days, IQR 3–11, we repeated tests after 5–7 days, depending on clinical indications).

No difference in the effects of CP administrations were observed in the children receiving transfusion within 7 days from the onset of symptoms, and the group who obtained the therapy after a longer period of time (Fisher’s exact test, *p* = 0.197).

**Figure 1:**
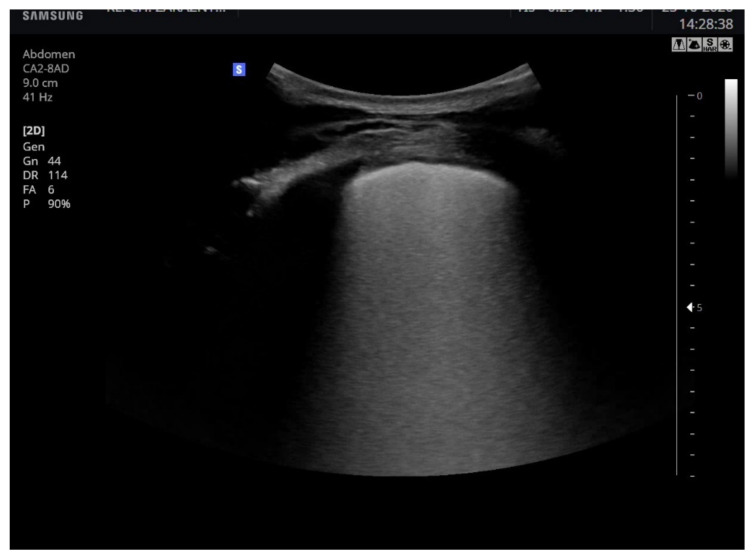
Lung ultrasound demonstrating areas of “white lung”.

We found abnormalities in the lung ultrasound examination (No. 4, 5, 8, 11 and 12) of the multiple B-lines type in five patients (ranging from focal through diffuse with areas spreading to the “white lung” appearance, Figure 1). In the same patients, parenchymal changes were described on the chest X-ray. One patient (No. 8), in addition to the abnormalities described above, had subpleural consolidations with pleural effusion (up to 32 mm in thickness). Chest-computed tomography was performed on patients No. 4, 8, 9, and 11. The changes described included: ground-glass opacities of varying intensity, from single, discrete changes (No. 8), through subpleural in several segments (No. 12) to diffuse, massive, bilateral with thickened bronchial walls (No. 4). In a patient with suspected lymphoma, apart from the apparent mass of the tumour in the mediastinum, radiologists described small parenchymal changes in the right posterior basal segment.

In all but one cases, the anti-SARS-CoV-2 antibody titer in CP was >1: 550. After CP transfusions, we did not observe any adverse reactions in the described children.

## 4. Discussion

The use of convalescent plasma as a treatment method has a relatively long history in medicine. Starting with the Spanish flu epidemic in 1918 [19], and more recently, during the SARS epidemic in 2003 [20], and the Ebola virus outbreak in Guinea in 2015 [21]. Based on reports from studies in the adult population, the use of CP reduced mortality in about half of the cases (meta-analysis; OR, 0.44; 95% CI, 0.25 to 0.77). An improvement in the clinical condition of adult patients was also observed compared to the group that was not treated with CP (OR, 2.06; 95% CI, 0.8 to 4.9, without statistical significance) [22]. In another systematic review, the conclusions on reducing mortality after using CP were ambiguous [23].

There are limited data on the pediatric population. In a case report of a child with congenital spherocytosis and X-linked agammaglobulinemia, the authors used CP due to slight improvement after supportive treatment. The boy recovered 6 days after the transfusion [24]. In a 4-year-old girl with acute lymphoblastic leukaemia (a severe course risk factor), a significant improvement in the clinical condition and a reduction in oxygen-dependent therapy were obtained after CP transfusion [25]. Among children with hematologic diseases, a case of a 9-month-old girl with juvenile myelomonocytic leukaemia, in whom tocilizumab was used in addition to CP, was also reported and improvement in her pneumonia was achieved [26]. In our centre, we considered CP as a possible therapeutic option in a girl with aplastic anaemia, probably based on COVID-19 (case included in the group of patients presented in the manuscript) [27]. American authors achieved a slightly less spectacular improvement in four critically ill patients with acute respiratory failure [28]. The combination of CP and remdesivir therapy was used in a 9-week-old infant with trisomy 21 and congenital heart disease, in whom COVID-19 exacerbated circulatory and respiratory failure. The authors recovered the described child [29]. In an 11-year-old girl without chronic diseases, CP was used because of its severe course: toxic-like syndrome and multi-organ failure syndrome. Rapid clinical improvement was achieved [30].

Although the results in the described cases are promising, conclusions regarding the effectiveness of CP should not be drawn basing on individual case reports. American authors obtained a very good clinical result in a group of four children with acute respiratory distress syndrome (ARDS). They achieved the best clinical improvement in the child who received the CP with the highest antibody titer, but the final antibody response of the patient did not differ significantly from the other children [31].

The main indication for the use of CP in our patients was the severe course of the disease due to underlying chronic conditions. We observed a broad spectrum of chronic diseases concerning the nervous, urinary, hematopoietic and cardiovascular systems. In two cases (No. 6 and 10), typical symptoms of COVID-19 were not observed. The indication for the use of CP in a patient with anorexia nervosa was the long-lasting presence of viral genetic material in a nasopharyngeal swab. The related difficulty in psychiatric care and the need for isolation was poorly tolerated by the patient. In the case of a 6-month-old girl (No. 10), who was admitted to our department after thermal burn with hot water, due to the observed abnormalities in the coagulation system (including prolonged prothrombin time) and percutaneous protein loss, a fresh-frozen plasma transfusion was planned. Due to the detection of the SARS-CoV-2 infection and the child’s severe general condition, we decided to transfuse CP.

The use of remdesivir in two of our patients was based on the clinical severe condition and coexistence of serious chronic diseases—in one patient with cerebral palsy with spastic paresis, epilepsy and Chiari’s syndrome, in the other Friedreich’s ataxia and dilated cardiomyopathy. The Food and Drug Administration (FDA) recommendations were followed: children had to be at least 12 years old, and weigh ≥ 40 kg. Data on the use of RDV in a large population of children are lacking, and to date, reports have shown an improvement in the clinical condition of the studied group of younger children (median age 5 years; IQR 4 months-11.6 years, 4 out of 8 were infants) and no side effects [32]. In a descriptive study of the European population (582 children, with a median age of 5.0 years, IQR 0.5–12.0), authors used RDV in 17 patients (3%), its effectiveness was not assessed [33].

In addition to indications resulting from comorbid disease, glucocorticosteroids (low-dose dexamethasone) have been used in selected patients, mainly in cases of extremely severe course or respiratory tract involvement. The use of glucocorticoids is supported by studies in the adult population comparing glucocorticoids to placebo or usual care in patients with severe or critical COVID-19. In a meta-analysis involving a total of 1703 patients, a reduction in 28-day all-cause mortality was found (33% vs. 41%, OR 0.66, 95% CI 0.53–0.82) [34].

Among the known infection sources in the described group, home contacts were the most common, which is consistent with the available data [35]. The spectrum of symptoms in children infected with SARS-CoV-2 is wide [36,37]. One of the most common findings is increased body temperature, which is consistent with the observations in our group [38]. A cough was also a common symptom. No patient was diagnosed with anosmia and dysgeusia, symptoms quite characteristic of COVID-19 but relatively rare in children (about 1% of children aged 0–9 years and up to 10% in the group of 10–19 years) [39]. In the results of laboratory tests, the typical symptoms were leukopenia and lymphopenia (approximately 17% and 13%, respectively) and elevated CRP levels, which is consistent with our observations [40]. Reports on the elevation of LDH activity are also covered in our data [41].

Typical changes in the ultrasound examination in COVID-19 patients include subpleural consolidations and individual or confluent B-lines–hyperechoic vertical lines departing from the pleural line and moving with it [42]. They testify to interstitial changes. Due to the radiological protection and good ultrasound sensitivity, the chest X-ray, or CT, was performed only in clinically justified cases. Such changes were seen in five patients from the observed group; in the remaining patients, the ultrasound image of the lungs was normal. The observed abnormalities in the CT examination were comparable to those described in the available reports, as characteristic of COVID-19: peripheral or subpleural round-glass opacities, inter-and intralobular septal thickening [43]. The presence of pleural effusion was most likely an expression of complications from the disease.

In the available reports, it is believed that the effectiveness of CP is inversely proportional to the time from the onset of symptoms to the time of transfusion. According to data on the adult population in the United States, the effectiveness of CP is greatest when the transfusion is performed within the first 3 days of hospitalisation [44]. The authors of the studies on the Polish adult patient population reached similar conclusions. They analysed the severity of the clinical course and the length of hospitalisation of patients who received CP ≤7 days and >7 days from the time of diagnosis. Still, they emphasised the limitations of the study, especially the small research group [45]. To date, we do not have data on children. In the group described by us, no statistically significant differences in the course of the disease depending on the time of implementation of the therapy were found.

It is worth emphasising that each medical procedure, including CP transfusion, carries the risk of adverse events (AEs). AEs can be classified into three groups: donor-related (e.g., hypotension, allergy, thrombophlebitis), equipment-related (e.g., thrombus formation, air embolism, bacterial contamination, citrate toxicity) and recipient-related (e.g., fever, chills). The characteristic complications after transfusion of blood products include transfusion-related acute lung injury (TRALI) and transfusion-associated circulatory overload (TACO) [46]. No side effects were found in patients treated with CP in our department, which is consistent with the majority of available reports on this therapy [8,44,45].

Eventually, we achieved clinical improvement in all patients. In patients with RT-PCR negativity after more than 14 days, it is hard to distinguish the influence of CP infusion and the natural course of the disease. 

The study includes a small group of patients with COVID-19, leading to difficulty obtaining statistical significance. Further research, especially RCTs, on the effectiveness of CP in treating COVID-19 in children is undoubtedly needed. There are currently clinical and preclinical trials of CP treatment in COVID-19, including children [8]. At the end of 2020, a preliminary protocol for the meta-analysis of the effectiveness of plasma therapy in children was published [47]. We hope that the results of the observations presented by us will contribute to further research on the treatment of the SARS-CoV-2 infection.

## Figures and Tables

**Table 1:** A collective description of the study group.

No.	Sex	Age (yr)	Comorbidities	CP Anti-SARS-CoV-2 Titer	Symptoms Onset to Admission (Days)	Admission to Transfusion (Days)	Length of Hospital Stay (Days)	Negative PCR (Days after Transfusion)	Anti-Inflammatory Treatment	Chest X-ray	Disease Severity (Oxygen Demand), Outcome
1	F	6	aplastic anaemia	1:600	3	37	59	3	DEX 0.3 mg/kg/24h	normal	Mo, T*
2	F	17	ADEM	1:1850	2	10	20	11	MPRED 30 mg/kg/24h	normal	Mo, R&D
3	M	14	CKD. KTx	1:600	2	12	20	6	PRED 0.1 mg/kg/24h	normal	Mo, R&D
4	M	12	CP. epilepsy. Chiari malformation	1:250	5	18	35	8	DEX 0.15 mg/kg/24h	parenchymal consolidations	S (FiO_2_ max 0.4), R&D
5	M	17	CP. epilepsy	1:650	5	7	31	8	DEX 0.15 mg/kg/24h	parenchymal consolidations	S (FiO_2_ max. 0.8), R&D
6	F	16	anorexia nervosa	1:600			33	3		normal	Mi, R&D
7	F	11	nephrotic syndrome	1:700	0	5	17	11	PRED 1.7 mg/kg/24h	normal	Mo, R&D
8	M	15	non-compaction cardiomyopathy	1:1700	2	6	9	3	DEX 0.15 mg/kg/24h	parenchymal consolidations	Mo (face mask O_2_ flow 6 L/min), R&D
9	M	8	suspected lymphoma	1:650	5	6	8	3		enlarged mediastinal shadow	Mo, R&D
10	F	6/12	thermal burn	1:700			8	3		peribronchial consolidations	Mi, R&D
11	F	1	sepsis. HUS	1:1600	2	3	22	3		parenchymal consolidations	S (FiO_2_ max 0.35), R&D
12	M	17	FA. DCM	1:600	7	10	17	11	DEX 0.15 mg/kg/24h	parenchymal consolidations	Mo (face mask O_2_ flow 7 L/min), R&D
13	F	5/12	renal agenesis. vesicostomy. VSD	1:550	0	3	15	30		normal	Mo, R&D

CP—convalescent plasma, PCR—polymerase chain reaction, ADEM—acute disseminated encephalomyelitis, CKD—chronic kidney disease, KTx—kidney transplant, CP—cerebral palsy, HUS—hemolytic uremic syndrome, FA—Friedreich’s ataxia, DCM—dilated cardiomyopathy, VSD—ventricular septal defect, PRED—prednisolone, MPRED—methylprednisolone, DEX—dexamethasone, Mi—mild, Mo—moderate, S—severe, R&D—recovered and discharged, T*—transferred to another hospital (patient required hematopoietic stem cell transplantation).

**Table 2:** A comparison of selected laboratory parameters before and after convalescent plasma transfusion.

	1 Day before CP Transfusion	3 Days after CP Transfusion
Body Temperature [°C]	38.0 (36.6–38.5)	36.6 (36.5–36.7)
WBC [×10^9^/L]	8.85 (5.18–12.6)	11.05 (4.77–11.9)
HGB [g/dL]	12.6 (9.8–13.7)	11.3 (10.2–12.6)
Platelets [×10^9^/L]	216 (152–285)	247 (216–295)
Neutrophils [×10^9^/L]	6.21 (2.66–9.43)	5.62 (2.72–7.93)
Lymphocytes [×10^9^/L]	1.52 (0.91–2.26)	1.91 (1.52–2.87)
APTT [s]	30.7 (28.9–32.8)	26.2 (25.7–29.1)
INR	1.17 (1.13–1.42)	1.1 (1.02–1.28)
Fibrinogen [N: 180–350 mg/dL]	275 (203–353)	249 (189.5–390.5)
D-dimer [N: <0.55 mg/L]	2.12 (0.65–4.62)	1.32 (0.74–2.44)
CRP [N: <0.5 mg/dL]	1.39 (0.14–3.46)	0.38 (0.02–6.39)
ALT [N: <39 IU/L]	16 (9–45)	38 (17–43)
AST [N: <47 IU/L]	30 (17–50)	20 (17–36)
LDH [N: 110–295 IU/L]	285 (244–465)	n/a
CK [N: <154 U/L]	81 (37–233)	122 (95–149)
Ferritin [N: 15–300 ng/mL]	173 (128.5–441.5)	n/a
BNP [N: <100 pg/mL]	128.1 (57.3–207.1)	136.75 (97.7–175.8)
IL-6 [N: <7 pg/mL]	6.4 (1–11)	n/a

Data points are indicated as medians (IQR). N—normal values, WBC—white blood cells, HGB—hemoglobin, APTT—activated partial thromboplastin time, INR—international normalized ratio, CRP—C-reactive protein, ALT—alanine aminotransferase, AST—aspartate aminotransferase, LDH—lactate dehydrogenase, BNP—B-type natriuretic peptide, CK—creatine kinase, IL-6—interleukin 6, n/a—not available.
